# Does ORTO-15 produce valid data for ‘Orthorexia Nervosa’? A mixed-method examination of participants’ interpretations of the fifteen test items

**DOI:** 10.1007/s40519-020-00919-2

**Published:** 2020-05-22

**Authors:** Elina Mitrofanova, Elizabeth Pummell, Laura Martinelli, Andrea Petróczi

**Affiliations:** 1 School of Life Sciences, Pharmacy and Chemistry, Kingston University, Penrhyn Road, Kingston upon Thames, KT1 2EE Surrey UK; 2 Peter Symonds College, Winchester, SO22 6RX Hampshire UK

**Keywords:** Orthorexia, Clean eating, ORTO-15, Psychometrics, Eating disorder, Pathological eating

## Abstract

**Purpose:**

Orthorexia Nervosa (ON) is defined as a pathological eating behaviour stemming from being “healthy” or “pure”. Survey-based studies typically rely on the ORTO-15 questionnaire or its variations to detect orthorexia. However, frequent post-hoc adjustments to the ORTO-15 suggest psychometric problems. In this study, we explored people’s cognitions about the ORTO-15 items to (1) identify problems specific to ORTO-15 items and (2) explore participants’ understanding of ON symptoms.

**Methods:**

Fifty adult participants (40% male, mean age = 34.0 ± 14.4 years) completed the ORTO-15, the Eating Attitudes Test (EAT-26) and the Obsessive–Compulsive Inventory–Revised edition (OCI-R). Qualitative data were collected using the modified “think aloud” protocol, which asked participants to ‘verbalise’ their responses to the ORTO-15 items. These qualitative responses were first analysed conjunctively with the quantitative responses; then subjected to thematic analysis.

**Results:**

ORTO-15 identified 64% of the participants for orthorexic tendencies. In most cases (76%), participants reported no issues completing the ORTO-15. However, in some cases, qualitative responses differed from quantitative ones. When people encountered problems, it was because of poor psychometric construction: lack of clarity, ambiguous wording and multiple statements in a single item. Elaborations around the ORTO-15 items formed four major themes: “preoccupation with physical appearance”, “control”, “food is fuel” and “alone, not isolated”.

**Conclusion:**

Even though in the majority of cases there were no issues with completing ORTO-15, thematic analysis revealed several discrepancies between our participants’ perceptions of the ORTO-15 items and the previously proposed diagnostic criteria for ON. The results suggest that ORTO-15 is, at best, a mediocre screening tool for ON, which is sensitive to diet but fails to have sufficient level of specificity to detect the pathological stage. More accurate instruments are needed to further research on ON.

**Level of evidence:**

V (cross-sectional descriptive study with qualitative analysis).

## Introduction

Orthorexia nervosa (ON) has been described as a set of behaviours and beliefs characterised by an obsession with “healthy” or “pure” eating [1]. This fixation on the purity of food as opposed to its quantity is the main feature of ON. According to the proposed diagnostic criteria by Dunn and Bratman [2], individuals suffering from ON are preoccupied with either affirmative or restrictive dietary practices believed to promote health. Dietary restrictions escalate over time and may cause the exclusion of entire food groups. Violation of self-imposed rules causes a sense of personal impurity, anxiety, and guilt resulting in compensatory behaviours such as an even stricter diet, exercise, or a “cleanse” (attempt at ridding the body of substances perceived to be toxic or unhealthy, often by limiting food consumption to only water or other liquids). Such behaviours may result in unbalanced and insufficient diets, weight loss, and impairment of social and professional lives. Individuals suffering from ON may have difficulty eating with others who do not share their rigid dietary beliefs, place a high value on maintaining control over food preparation and tend to follow a very strict mealtime schedule [3–5].

Despite the growing interest in ON in academia [2, 6], this condition is not officially recognised in the Diagnostic and Statistical Manual of Mental Disorders [7]. Some researchers suggest that ON is strongly related to obsessive–compulsive disorder (OCD) [8, 9], while others suggest that symptoms of ON overlap with symptoms of Anorexia Nervosa (AN) [10]. Unlike individuals diagnosed with AN, individuals suffering from ON are not secretive about their preferred diet and do not experience body image disturbances that are based on perceived weight or body shape [2]. A neuropsychological study found symptoms of all three conditions (OCD, AN and ON) to be related [8]. However, it is not clear if obsessive thoughts are a source of distress for individuals suffering from ON, or if compulsive behaviours are aimed at preventing a catastrophic event or at reducing distress. Despite lacking official recognition, ON is a sufficiently recognised entity in need of further inquiry [11]. What makes ON an intriguing condition is that various definitions seem to capture part but not the whole essence of the phenomenon. Reflecting on developments since the inception of the term, Bratman [12] emphasises the progression of the condition where the first stage is more of a (commendable) lifestyle choice adhering to a healthy diet (and exercise), even if such diet involves unusual and irrational ideas. It is the second stage of ON that is problematic and involves pathological behaviour.

### Detecting orthorexia nervosa

Despite that the condition has not been recognised as a disorder, the literature on orthorexia has been dominated by the studies aimed at establishing its prevalence in a number of different samples [13–17]. The absence of official recognition and established clinical diagnostic criteria of the condition renders prevalence assessment premature. Firstly it is because the presence of a condition cannot be detected without having a clean definition of what is being assessed. Secondly, even if there is a general agreement that the condition exists, the absence of established diagnostic criteria for ON impairs the development of screening tools for the condition, and limits the validity of prevalence assessments.

To date, two questionnaires are commonly used to measure the prevalence of orthorexia: the 10-item Bratman Scale [4] and the ORTO-15 questionnaire [13]. The academic community has mostly disregarded and criticised the 10-item Bratman Scale for the lack of validity demonstrated in the research, and for the fact that creators of the scale did not follow standardised statistical procedures when creating it [18].

The ORTO-15 questionnaire, which has been the most widely used measure [19], consists of 15 multiple-choice questions, six of which were taken from the Bratman scale. There are several translations of the original Italian version of ORTO-15, including Turkish, Hungarian, English, and Polish. Responses are scored on a 4-point Likert-type scale, which includes: “always” = 1, “often” = 2, “sometimes” = 3, and “never” = 4. Scores above 40 are suggested to indicate the absence of ON. According to the original authors’ instructions, items 2, 5, 8 and 9 are reverse-scored (“always” = 4, “often” = 3, “sometimes” = 2, “never” = 1). Items 1 and 13 are scored as: “always” = 2, “often” = 4, “sometimes” = 3, “never” = 1.

### Concerns about ORTO-15 as a screening tool for orthorexia

The results from prevalence studies using ORTO-15 vary from 6% prevalence in an Italian sample to 88.7% in a group of female nutritionists [2]. Interestingly, a recent study with US college students found a prevalence of 71%, although less than 1% experienced impairment in everyday activities and medical problems caused by their diet [20].

Recognising potential problems with ORTO-15, Moller and colleagues conducted confirmatory factor analyses of the 15-, 11- and 9-item versions of the scale and concluded that none of the three versions showed acceptable model fit [21]. With eliminating two items from the shortest scale, the ORTO-7 model was proposed. Items of the different ORTO-scale variants are presented in Table 1.

**Table 1:** Summary of qualitative elaborations on each of the ORTO-15 items in the context of different English variants

ORTO-15 items	ORTO-11 version (Arusoglu et al. 2008)	ORTO-9 version (Missbach et al. 2015)	ORTO-7 version (Moller et al. 2018)	Qualitative analysis (Problematic items)
1. When eating, do you pay attention to the calories of the food?	–	–		13
2. When you go in a food shop do you feel confused?	–	–	–	15
3. In the last three months, did the thought of food worry you?				17
4. Are your eating choices conditioned by your worry about your health status?				16
5. Is the taste of food more important than the quality when you evaluate food?			–	21
6. Are you willing to spend more money to have healthier food?			–	10
7. Does the thought about food worry you for more than three hours a day?				12
8. Do you allow yourself any eating transgressions?		–	–	7
9. Do you think your mood affects your eating behaviour?	–	–		6
10. Do you think that the conviction to eat only healthy food increases self-esteem?			–	19
11. Do you think that eating healthy food changes your life-style (frequency of eating out, friends, …)?				13
12. Do you think that consuming healthy food may improve your appearance?			–	7
13. Do you feel guilty when transgressing?		–		12
14. Do you think that on the market there is also unhealthy food?		–	–	9
15. At present, are you alone when having meals?	–		–	4

Although new instruments for detecting orthorexia emerged in Germany, (Düsseldorf Orthorexia Scale [5]), USA (Eating Habits Questionnaire [22]), Spain (Barcelona Orthorexia Scale [23] and Teruel Orthorexia Scale [24]), the ORTO-15 has remained the most widely used scale in the academic literature on ON, thus warranting the need for further investigation to ascertain if items of ORTO-15 fully capture the construct of orthorexia. Yet, and despite the recurrent and well-documented problems with ORTO-15 [25, 26], no attempt has been made to explore the potential reasons for the poor performance.

### Aim

This study aimed to investigate the reasons behind the poor performance of ORTO-15 with the view to identify ways for improvement and to facilitate developing new screening tools. Initially, our study aimed to explore people’s thought processes about the ORTO-15 items. In line with the “think loud” methodology, we set out to understand why certain items are problematic. Putative reasons for this could include items where participants are unsure what the statement is about (e.g., contains two issues in one sentence) or have cognitive conflicts (e.g., honestly should answer affirmatively but for a different reason). This initial phase focussed on the functionality of ORTO-15.

Subsequently, we also analysed the qualitative responses to identify congruencies and potential discrepancies between participants’ experience of orthorexic tendencies (where applicable) and the existing understanding of the condition in the literature. This phase was conducted retrospectively via analysing participants’ thoughts expressed for each ORTO-15 item, not by directly asking participants to elaborate on their views on orthorexia. With this added analysis, we focused on the introspective reflection about the *behavioural aspects* with the view to investigate which facets of orthorexia, if any, manifest in people’s thoughts when responding to items of ORTO-15.

## Method

### Design

The study used a mixed methods design, incorporating both quantitative and qualitative methods. Participants first provided demographic, self-reported anthropometric and health-related information. Qualitative data consisted of participants’ written “think aloud” responses to the ORTO-15, which were analysed via content and thematic analyses.

In the qualitative component of the study, we asked participants to reflect on and verbalise their thoughts when completing the ORTO-15. We employed a method inspired by the “think aloud” protocol [27], which requires participants to verbalise their thoughts while completing a cognitive task. The “think aloud” method has proven to be a valuable way of exploring how and why respondents arrive at their answers, and to identify problems responders experience when completing a scale. It has been used successfully to examine the content validity of several questionnaires [28–33]. Successful utilisation of the “think aloud” method may, therefore, offer empirical support for improving psychometric measures. This study’s method deviated from the original “think aloud” protocol in two ways: participants’ thoughts were captured retrospectively not simultaneously; and in written form not verbally. The current procedure involved recording people’s written verbalisations of cognitive processes in response to every item of the ORTO-15. The advantage of conducting retrospective “think aloud” protocol involves a decrease in reactivity whereby performance might be enhanced due to a more structured working process or diminished by a double workload of responding to a question and vocalising the thought process simultaneously. Participants are allowed to provide reflections on the items at their own pace.

Given the lack of understanding of the symptoms of ON, and the potential overlap with other eating disorders and OCD [10], this study has moved beyond single “think aloud” assessment and included additional psychometric measures to identify possible link to other disorders; and most importantly their potential influence on how people answer the ORTO-15 items. Thus the quantitative part included two established psychometric measures designed to identify the presence of OCD symptoms and to assess eating disorder risk. All collected data were collected anonymously, with implied consent. Qualitative responses were entered by the participants directly onto the online survey. Although participants were recruited via personal contacts, there was no way researchers could tell who accepted the invitation and completed the survey because identifiable personal information (including IP addresses) were not recorded.

### Collection of the sample

Adults residing in the UK with a minimum age of eighteen years old were invited to participate in this study. No exclusion criteria were applied to ethnic background, occupation or sociodemographic status. Individuals had to be able to speak English fluently as a second language or be native English speakers. Participants were recruited from the research team’s contacts using the snowball sampling and were approached based on the research team’s prior knowledge of existing restrictions in their diet. Several individuals (informants) known to exhibit orthorexic tendencies (i.e., restricted eating behaviour, avoidance of certain foods, particular food beliefs) were approached and asked to participate in this study voluntarily and to help identify individuals known to them that exhibit similar eating patterns. Our purposeful sampling strategy targeted people who were interested in integrating ‘clean eating’ principles into their daily life; interest; and reported at least some signs of orthorexic eating behaviour. These included self-imposed distinctive and sustained dieting behaviour for health reasons; voluntarily restricted their food based on characteristics of the foodstuff (i.e., omitted certain food groups for no medical reasons; or only consumed specific type of food such as organic, raw, etc.). Because the ORTO-15 is designed to screen population for orthorexia, we included a wide spectrum of ‘healthy eaters’, potentially problematic and non-problematic, to see if responses to the ORTO-15 items differ between those who score beyond the recommended cutoff of 40.

### Measures

All questionnaires were hosted on a closed survey platform (SurveyMonkey) accessible via a designated link. Demographic information (age, gender, ethnicity, occupation, and current living situation) was collected. Self-reported anthropometric measures consisted of height, current weight, lowest weight, highest weight, and desired weight. Health-related questions enquired about the presence of diagnosed health conditions that might affect eating behaviour.

#### 2.3.1 ORTO-15

The English version of ORTO-15 included 15 original items with a comment box for each question. Responses were scored in accordance with the original authors’ instructions. According to the authors of scale, scores beyond 40 showed a good predictive capability for the presence of ON [13].

#### Psychometric measures

The Eating Attitudes Test (EAT-26). The EAT-26 [34] is a widely used 26-item standardised self-report screening tool used for identifying symptoms characteristic of eating disorders. It consists of three subscales: (1) dieting, (2) bulimia and food preoccupation, and (3) oral control. A score higher than 20 suggests the possible presence of disordered eating [34].

The Obsessive–Compulsive Inventory-Revised (OCI-R). The OCI-R [35] is an 18-item self-report measure for assessment of six common OCD symptoms: checking, hoarding, obsessing, ordering, neutralising and washing. Scores above 20 indicate presence of OCD [35].

### Procedure

Participants were asked to voluntarily take part in the study by completing the online questionnaire and to set aside one hour to comfortably complete all steps. They were made aware that voluntary completion of all measures implied their consent. As part of the recruitment, participants were briefed verbally, and an information sheet was provided as an embedded part of the questionnaire. Participants were then asked to complete the English version of ORTO-15. The following instructions were provided at the top of the page:

After reading the question, select one response from the prescribed list (i.e. “always”, “often”, “sometimes”, or “never”) and then explain the selection that you made in the comments box provided. Ensure that you have fully answered a given question before moving on to the next.

Additionally, the following instructions were presented before each item of the scale:

Please explain why you answered the way that you did (try to be specific, give an example if needed). We would also be interested to know the extent to which you believe that the response you selected accurately reflects your thoughts, feelings and/or behaviours relevant to the question. You may also want to highlight any terms in the question that are confusing or ambiguous.

The comment boxes were inserted to capture participants’ thought processes for qualitative analysis.

### Data analysis

Quantitative statistical analyses were performed using IBM SPSS (Statistical Package for Social Sciences) Version 24.0 and JASP (Version 0.11.1) computer software. Bivariate correlation coefficients (*r*) between EAT-26, ORTO-15 and OCI-R were calculated using the Spearman formula. Associations between categorical variables (binary status of disordered eating, OCD and ON) were tested using chi-square statistics with Fisher’s exact probability. Internal consistency reliability of EAT-26, OCI-R and ORTO-15 scores for this sample are expressed as Cronbach’s alpha coefficients and McDonald’s omega. Participants’ open-ended explanations for their response to the ORTO-15 items and feedback about the clarity of the instrument represent the qualitative data. The first author conducted a content analysis [36] of participants’ feedback using the Atlas.ti software to identify any problems participants encountered when responding to the ORTO-15. Taking into account both the think-aloud protocols and the quantitative responses to the ORTO-15 a full coding frame was developed. Codes were based on the discrepancies and difficulties expressed while completing the questionnaire. The coding frame was then applied to all the data. Ten transcripts, chosen using the random number generator, were coded by the second author. The initial between-coder agreement was 85%. The coding frame and the coding were revised after the discussion between the two authors and the agreement increased to 100%. The final version of the coding frame consists of five codes where the fifth code represented “no problems” (indicating there were no problems experienced when responding to an item). The remaining four codes represent problematic responses. The coding frame with codes’ definitions can be found in Table 2.

**Table 2:** Coding frame

Code and definition	Description	Example quotes from participants
1. Questioned the wording of the item	Participants did not understand and criticised specific words in the questions. This issue had appeared 21 times across ten items when participants expressed doubts about how the questions were worded. Most often this issue appeared in response to item 4 of the ORTO-15. Individuals openly admitted to not understanding some of the words used in ORTO-15	P16 in response to item 4: When I feel overweight and unhappy with my body image, this motivated me to eat healthier. It particularly works when I start to see a physical change. The question is a little confusing as some people have a different interpretation of a ‘health status’ P49 in response to item 8: Transgressing is the wrong word, the implication that my food rules are law is rather extreme. Also, not everybody understands the meaning of transgressing P18 in response to item 8: I find this question ambiguous, it’s not a word I associate with eating. I guess it means going beyond the eating limit?…
2. Did not understand the meaning	When participants did not understand the meaning of the question, provided statements unrelated to the item, and questioned the sensibleness of the item. This problem came up 79 times across all the items when participants struggled to understand what the question was asking. This issue was especially pronounced across items 2 and 10	P29 in response to item 7: Don’t really understand the question P36 in response to item 2: I don’t understand what there is to be confused about! P37 in response to item 14: This question is slightly confusing to understand, but I do think that there is a lot of unhealthy food available, and sometimes this is dressed up as being good for you. However, I am not entirely sure what this question is asking P1 in response to item 5: Hard question…something that tastes good is not necessarily quality, depending on how you measure it.” and “all of this is more complicated than can be answered here… P26 in response to item 2: What do you mean by more money? More than I usually spend? Healthier than what?…
3. Agreed only to a part of the question	When participants’ answers indicate partial agreement or disagreement with the question often based on additional information, conditions, or situations. This issue appeared 46 times across the data. Most often it was observed in item 5. Participants expressed their agreement or disagreement with the question based on additional reasoning suggesting that they applied their own frame of reference influenced by alternatives created by the individuals to answer this question	P3 in response to item 12: I think that consuming healthy food can improve your appearance if it is eaten in the right portion sizes… P4 in response to item 12: Eating healthy has been proven to clear skin, but again it’s like a placebo, although it works, it doesn’t happen overnight P43 in response ot item 5: Sometimes when I’m cooking my own meal I don’t worry about what I’m putting in it, I just do what will make it taste better, but if I was out buying food that’s when I calorie-count
4. Qualitative response did not concord with quantitative response to questionnaire item	When there is a difference between the reasoning in participants’ comments and their response in the questionnaire. This issue has appeared 35 times across 13 items with the most comments clustering in item 3	P18 chose “never” as a quantitative response to item 3 while the qualitative comment indicates the opposite to be true: “When I am consciously eating healthy I always worry about my food choices. Especially when having a good social life, it includes a lot of bad food and drink choices which makes me worry about food” P41 also chose the quantitative response “never” while the qualitative comment indicated: “I'm always worried about what to eat due to my weight issues”
5. No problem	When participants did not encounter any difficulties answering the question	

The first author conducted thematic analysis procedure as defined by Braun and Clarke [37] to identify whether participants’ feedback was related to orthorexic symptomatology. After repeated reading, the “think aloud” transcripts were explored using open thematic coding according to the “bottom-up principle”. The coding involved assigning codes to the data based on the semantic and conceptual readings. The next steps involved searching for subthemes by means of revisiting the codes and searching for the meaningful patterns across the data that later were grouped into themes. The emerged themes and subthemes were discussed and agreed upon during meetings between the authors.

## Results

### Description of the sample

Initially, 66 individuals took part in this research. Eight individuals provided demographic information but did not complete any other measures. Their data were removed from the final analysis. Another eight participants reported having medical or psychological conditions that may have an impact on their eating behaviour (i.e., depression, Irritable bowel syndrome, bulimia, anxiety), and their data were therefore excluded. The final sample consisted of 20 males and 30 females, mean ages of 34 years (SD = 16.3) and 35 years (SD = 13.2), respectively. The majority were of White British descent (88%), and over half (52%) lived with a partner, with an additional 30% living with parents. The average BMI was 25.3 kg/m^2^ (SD = 6.9).

### Quantitative analysis

#### Descriptive statistics

The mean score for the ORTO-15 was 37.82 (SD = 4.19) with 64% of the sample scoring in the ON range. The OCI-R mean score was 12.14 (SD = 9.65), and the EAT-26 mean score was 10.08 (SD = 8.99), indicating that generally, the sample had a healthy eating attitude. Seven of the 50 participants (14%) were identified as being potentially at risk for disordered eating. Eight participants (16%) were identified for showing OCD tendencies.

#### Internal consistency reliabilities

The internal consistency reliability of the scores of ORTO-15 in this study was found to have a Cronbach’s alpha score of 0.47, which is considered to be very low. McDonald’s omega coefficient was 0.56.

The Cronbach’s alpha value of 0.88 for the OCI-R was calculated from this study’s sample which signals a good internal consistency reliability of the scores of scale. McDonald’s omega coefficient was 0.89.

The Cronbach’s alpha value for the scores of EAT-26 in the present study was 0.86, also indicating good internal consistency reliability. McDonald’s omega coefficient was 0.85.

Omega coefficients are interpreted in the same manner as Cronbach’s alpha. The difference between the observed alpha and omega coefficients lies in the models that define alpha (essential tau-equivalence) and omega (congeneric). In this study, the discrepancy between alpha and omega coefficients of ORTO-15 may have resulted from the violation of essential tau-equivalence model (the assumption of error score of any pair of items is uncorrelated). If this assumption is violated, the true reliability is underestimated [38].

#### Associations

Key findings from the correlation analysis were as follows: ORTO-15 score was significantly and negatively correlated with the EAT26 score (*r* = − 0.66, *p* < 0.001) and the OCI-R score (*r* = − 0.30, *p* = 0.03). Furthermore, a statistically significant positive correlation was observed between the EAT26 and OCI-R test scores (*r* = 0.33, *p* = 0.02). (Note that ‘at risk’ status is indicated by high scores on EAT-26 and OCI-R but low scores on ORTO-15, which explains the negative correlation).

### Qualitative analysis

#### Content analysis on the functionality of ORTO-15

Content analysis of the “think aloud” responses revealed that participants did not encounter problems while filling in the ORTO-15 for the majority of the time (76%). However, a total of 179 problems were identified. Responses were classified as “no problem” unless there were “think aloud” data to the contrary. The mean number of problems per participant was 3.44 with a range of 0 to 9. The coding frame, the definitions of the problems and the quotes from participants are presented in Table 2, while Table 3 presents the frequency distribution of the identified problems.

**Table 3:** Frequencies of codes distribution

Codes	Items														
	1	2	3	4	5	6	7	8	9	10	11	12	13	14	15
1	0	1	2	4	2	1	3	2	0	1	0	0	3	2	0
2	4	12	5	6	6	4	5	3	3	13	4	2	5	5	2
3	6	0	0	3	10	3	2	1	2	4	6	5	2	2	0
4	3	2	10	3	3	2	2	1	1	1	3	0	2	0	2
5	38	35	33	34	29	40	38	43	44	31	37	44	38	41	46

Content analysis revealed the item that participants had the most problems with was item 5 (Is the taste of food more important than the quality when you evaluate food?) Most often participants suggested alternative reasoning that the taste is better if the food is of good quality and overall, the two concepts are inseparable. Item 15 (At present, are you alone when having meals?) was the item that elicited the fewest issues. Participants offered comments in a “yes” or “no” format without further elaboration. Most individuals gave an affirmative answer to item 14 (Do you think on the market there is also unhealthy food?). However, the endorsement of this statement does not always mean a higher likelihood of meeting the criteria for ON.

The scores of the scale demonstrated a very low coefficient of internal consistency reliability (*α* = 0.47) which is to be expected considering the number of problems identified. Participants struggled to comprehend the meaning of the item 2 “When you go in a food shop, do you feel confused?”. It was unclear to participants why going to a shop would elicit confusion. Another item that was met with a similar reaction is “Do you allow yourself any eating transgressions?”. Many participants did not know what the word “transgressions” meant. Understanding the question is the first step participants take when completing a questionnaire. To avoid variation in question comprehension researchers are advised against the use of ambiguous and unfamiliar words [39].

#### Thematic analysis on the behavioural aspects in ORTO-15 items

Participants’ elaborations went further than just simply identifying potential problems with ORTO-15. Therefore, thematic analysis of the transcripts was conducted to identify whether participants’ “think aloud” data are linked to the concept of ON and the proposed diagnostic criteria. Four themes were identified: “preoccupation with physical appearance”, “control”, “food is fuel”, and “alone not isolated”. Participants are identified by numbers and their respective scores on ORTO-15 are provided in brackets.

##### Preoccupation with physical appearance

For the majority of participants in this study, striving for a healthier diet was motivated by their desire to manage their weight. Participants mention an improvement in physical appearance as the factor that drove them to start eating healthier. Participants identify this improvement in physical appearances, such as weight loss or clearer skin as a direct cause of adherence to the self-imposed diet. These quotes from the transcripts illustrate the point: P1(38): “I have been trying to lose weight, so I was concerned about eating certain things…I wouldn’t say I was worried, but I was conscious of what I was eating.” P10(37): “The experience I have of this is that my skin looks better and keeping an eye on calorie content means I have more control over my figure and therefore appearance…” For many participants in our study weight loss has come to represent their ability to achieve health and well-being.

##### Control

The second identified theme can be defined in terms of participants’ perceived control over their eating behaviours and exercise routines. Participants reported having a strict routine that involved planning meals and regular exercise. People experienced negative emotions if the self-imposed routine wasn’t followed and tried to compensate for it by an extra workout or a stricter diet the next day. For example, P24(41) reported: “I feel guilt if I am not getting to eat in my usual healthy manner”.

Transgressions did not cause any adverse emotions if they were planned and incorporated into the diet. For example, in response to “Do you allow yourself eating transgressions?” P6(42) provided: “Yes small transgressions which I would call treats!” In response to item 13 (Do you feel guilty when transgressing?) P27(39) replied: “it’s a conscious decision, so it would seem illogical to me to then feel guilty. I would factor that into the decision itself.” In fact, by allowing themselves controlled deviations from their diets, participants reported a higher likelihood of adherence.

P47(39) described the role of these deviations: “For the long term, a small transgression avoids completely going off the rails and binging.” Planning served as a protective factor against worry, guilt, and provided a sense of being in control in social situations when participation involved consumption of alcohol and food thought to be unhealthy.

##### “Food is fuel”

This theme describes the participants’ relationship with food. The comments indicate how discourse about food has moved to a view of food as a source of “fuel” for maximising health or physical performance. For example, P41(34) expressed: “I prefer healthy food as then I know my body has the best fuel.” Participants believed that a healthy diet has a direct impact on psychological well-being and physical health:

“What you put into the engine determines how it runs. Again back to vitality. If you are always down, low energy and no get up and go, then the diet in most cases is the cause. Tiredness is the huge issue for women and men with young families, so high energy and protein is important when you lack sleep. For most health issues if you can detect them early enough, food can make a marked difference.” (P31(34). P21(36): “I believe there is a connection between eating healthily and feeling good about oneself, physically and mentally. I know I’m more likely to engage in healthier activities and exercise when I’m following a healthy eating plan, which in turn increases the sense of well-being.”

Participants linked health as a central organising factor in their practices of food selection. A particular perception of the body as a machine that needs the best quality nutrients to perform at its best has emerged from the transcripts.

It appears individuals in this study were faced with a constant challenge to sort through the food-related information and were preoccupied with the evaluation of risks and benefits of food. Participants demonstrated a high level of confidence in their knowledge of nutrition and defined their relationship to food as a never-ending process of information seeking and self-education potentially with a limited scientific basis. On the other hand, some individuals found this strive for nutritional knowledge very distressing and expressed uncertainty about the nutritional information they encounter on a day-to-day basis. P3(35) has expressed a general mistrust to food-related information offered on the market: “There are so many food items out there now that claim to be healthy or better for you but all with hidden sugars and salt. It can be very confusing to know what is best to eat and best to buy.”

##### “Alone not isolated”

This theme describes various social contexts within which participants described their food choices and practices. Impairment of social life resulted from an excessive focus on healthy eating has been implicated in one of the diagnostic criteria proposed by Dunn and Bratman [2]. This study, however, did not yield support for this assumption. Participants did report being alone during meal times which was not experienced as social isolation but was rather a conscious preference or reflected individuals’ living situations:..P46(37): “I live alone so yes am always on my own when I eat breakfast and dinner, lunch at work.”

P38(35): “Monday to Friday I have lunch at work I bring food from home cooked by me the night before I usually eat with colleagues. Evenings and weekends I eat with my husband.” The importance assigned to following a healthy diet outweighs the need for social interaction. Furthermore, some individuals perceived social engagements as an obstacle to a healthy lifestyle:

P17(31): “I feel like my social life always gets in the way of eating healthily. If I am eating healthily, I am less likely to go out and have a social life as I become too tempted to eat the wrong foods…”.

Even though the data suggest that participants’ social lives were affected by their diets, psychological discomfort, proposed by previous research, caused by social isolation was not reported in this study.

## Discussion

The main purpose of this study was to explore the nature and extent of problems individuals encounter when they complete the ORTO-15. This study also sought to compare participants’ responses to ORTO-15 with three additional questionnaires measuring related phenomenon to further determine the validity of the ORTO-15. As in previous studies employing the “think aloud” technique [28, 31], participants did not encounter any difficulties responding to the scale the majority of the time. The success of the think-aloud technique depends on participants’ ability to verbalise their thoughts, and individuals differed in their performance throughout the task. Because the responses were coded as “no problem” unless “think aloud” data indicated otherwise, it is important to acknowledge that the issues with the scale might have been underestimated.

### Prevalence of ON by ORTO-15

Consistent with previous studies using the ORTO-15, the putative prevalence of ON symptoms was relatively high (70% of the sample) compared to the prevalence of AN in general population (0.9% among women and 0.3% among men) [40]. Similar findings were previously observed in other countries where researchers used the ORTO-15 to assess the prevalence of ON in various populations [15, 18, 41, 42]. However, reported ‘prevalence’ data on ON must be interpreted with great caution for multiple reasons.

Firstly, ON has not yet been recognised as a bona fide disorder, thus any assessment of the condition is somewhat arbitrary and based on assumptions and not clinical data about the aetiology and manifestation of ON (e.g., ON is a form of an eating disorder or an obsessive–compulsive disorder). Secondly, no studies using ORTO-15 were set up to estimate population prevalence. Without exception, these studies used convenience sampling not representative for the population [19]. At best, these studies show the number of individuals identified for reporting putative ON symptoms in the sample. Finally and most importantly for our study, the dominant assessment tool, ORTO-15, has been challenged for its validity and propensity to identify the healthy spectrum of controlled diet as ON which inflates the number of observed ON cases in the sample. Our study adds qualitative evidence to this criticism. It is also notable that those participants in this study who were identified for ON by the ORTO-15 scored just beyond the cut-off point of 40 thus they were borderline for ON. Using a more exclusive cut-off point to fall between 35 and 40 for being more specific in identifying ON tendencies (Table 4, p31 in Donini et al. [13]), these individuals would have classified as non-ON. Such choice of course reduces the chance for incorrect positive classification at the expense of an increase in missing genuine positive cases. Because ON is thought to be on a continuum [43], cut-off points should be interpreted in context, not in absolute terms.

### Construct validity and accuracy

This study found a significant negative correlation between the scores of ORTO-15 and both OCI-R and EAT-26. Lower scores on ORTO-15 indicate the presence of ON while higher scores on OCI-R and EAT-26 indicate the presence of OCD and eating pathology. Observed negative associations, therefore, suggest that there are overlaps between ON and symptoms of other eating disorders as well as OCD. The association between ORTO-15 and EAT-26, however, needs to be interpreted with caution since there is similarity between items in these questionnaires.

### Functionality of ORTO-15

Problems were identified across all items, and 46 out of 50 participants encountered at least one issue. Four individuals did not elaborate any ‘think aloud’ data but responded to the scale items. Their contributions were, therefore, coded as ‘no problem’. Items that elicited the biggest number of issues were: 5, 10, 3, 4, and 2. In a study by Moller and his research team [21], items 5, 2 and 10 were highlighted as problematic and dropped from the developed ORTO-7 as shown in Table 1. Item 15 elicited the least confusion. However, the wording of this question does not allow for the intended concept of social isolation to be identified as potentially causing distress. Even though the nature and frequency of the problems varied, all items elicited at least one issue.

### Orthorexic traits: comparing reflection on the behavioural components of ON with other disorders

Results of the thematic analysis in this study support the hypothesised overlap of obsessive–compulsive and eating disorder traits in ON. The identified “control” theme is a factor underlying participants’ adherence to self-imposed diets. Previous studies have recognised the importance of personal control in eating disorder symptoms and OCD [44]. For example, people suffering from OCD often perform strict monitoring of their thoughts and actions and impose rules to dictate their behaviour. Behaviours such as checking, hoarding and performing rituals may be understood as attempts at establishing control. What the participants in the current study described are very similar to the attempts at establishing control over one’s environment experienced by individuals suffering from OCD. Control has also been studied for its connection to AN [45]. Fairburn and colleagues, for example, proposed that within the AN framework being successful at controlling one’s body shape and weight is an indicator of self-worth and overall self-control [46]. Also, many individuals report beginning to diet at a time of their lives they perceived to be chaotic and beyond their control [47]. Results of this study suggest that control, despite being one of the symptoms implicated in AN and OCD, might be one of the main features of ON.

Despite literature suggesting that ON’s most pronounced difference from other eating disorders is the motivation for following a diet of choice, our data revealed that the desire to lose weight was a significant factor. In past research, weight loss as a behavioural motivator was linked with the symptoms of AN [48], while the lack of desire to lose weight is one of the most critical factors separating ON from other eating disorders [2]. Similar to this finding, a recent study investigating a possible link between ON, perfectionism, body image, and attachment style has identified that fear of becoming overweight and a greater focus on appearance to be associated with lower scores on the ORTO-15 [49]. Physical appearance as a main motivating factor for following a “clean” diet could have a bigger role in ON than previously suggested.

Another identified theme sheds light on participants’ social lives: the data suggest individuals did not place any importance on the social rituals surrounding food consumption. It may be that this phenomenon is experienced by society as a whole and does not indicate the presence of ON. Nicolosi [50] proposed that orthorexia as a concept can be extended beyond individual pathology to describe a social phenomenon. Nicolosi argues that individuals in modern society are constantly reminded of the importance of diet on their physical health while at the same time the distance between them and food production grows. People have less and less knowledge about how food is managed, processed, and sometimes prepared while the discourse about healthy eating in popular media intensifies. This lack of knowledge about food production and intense discussion about risks and benefits of a healthy diet is at the core of rising dietary anxiety and food risk perceptions [51]. In today’s society, family meals are often sacrificed for work responsibilities. For the participants, social isolation was not a cause for distress but rather a general aspect of changing social habits. It is possible that this phenomenon is a societal norm and not indicative of ON and therefore not valid in terms of diagnosis. Themes identified in this study suggest that ON might have more in common with AN and OCD than was previously suggested. In addition, some concepts (e.g. social isolation and a lack of consideration for one’s weight) did not seem relevant to the experiences of participants in this study.

### Limitations

Our study has its limitations, among which are those of the “think aloud” method. The “think aloud” protocol states that participants are meant to verbalise their thoughts while completing a scale, in this study the data were collected online which limits researchers’ supervision over the process. For future research, it would be beneficial to conduct in-depth interviews to explore people’s experience of ON and contribute to the creation of a reliable diagnostic tool. Another improvement would be to carry out a nutritional assessment of participants’ diets. Research in the field of ON is still scarce, and to date, there are no universally accepted diagnostic criteria. Without a proper dietary assessment, it is impossible to ascertain if the orthorexic diet does lead to malnourishment as some of the proposed diagnostic criteria claim. Future research should focus on developing a new diagnostic tool as well as investigate the nutritional composition of the orthorexic diet.

Another possible limitation to this research is the modified procedure of the “think aloud” protocol. Concurrent variation of the protocol might have provided a richer account of the potential issues with the scale. Non-verbal information (pauses, utterances, body language) that concurrent “think aloud” procedure provides could contribute to further understanding of the difficulties people experienced when responding to ORTO-15.

The second part of our study, which led to the thematic analysis of the qualitative responses from our participants, presents a post hoc analysis of the existing data. As such, results from this only offer limited insight into people’s thoughts on their choices about diet and eating habits, and not on ON. We conducted and included this secondary analysis because we felt that the qualitative data add value to this study and can inform future research on and screening measures for ON. The richness of these data is also limited by modified ‘think loud’ procedure.

## Conclusion

In conclusion, this study attempted to identify problems people experience completing the ORTO-15. We have conducted a “think aloud” protocol to address the issues with the scale. Thematic analysis of the data has brought forward aspects of ON previously overlooked in the research. The instrument’s validity was under scrutiny by earlier research, and our results highlight a number of problems with the ORTO-15. The ORTO-15 is not an adequate scale to detect orthorexic behaviours and attitudes. Taking the qualitative and quantitative results together, it appears that at best, ORTO-15 taps into diet habits and lifestyle (stage one) but fails to detect the pathological aspect (stage two). To date, several questionnaires have been developed. However, attempting to identify prevalence rates of a condition that is yet to be defined is at best premature. More effort should be directed at determining ON as a valid construct.

### What is already known on this subject?

ON has been recognised as a potentially pathological condition. There is a lack of agreement on the diagnostic criteria and tools. The commonly used tool to identify ON, ORTO-15, has been suggested to be problematic due to its poor validity and reliability. The reasons for the poor performance of ORTO-15 are yet to be specified.

### What does this study add?

This study scrutinised each item of ORTO-15 for functionality (clear statement and instructions; absence/presence of disambiguity, etc.) and content (putative behavioural indicator of ON). Problems were detected across all items. The problem with ORTO-15 lies in its accuracy: it is sensitive to identifying a peculiar dieting habit but lacks specificity (no differentiation between peculiar but normal eating vs. pathological condition).
